# PPARs: Protectors or Opponents of Myocardial Function?

**DOI:** 10.1155/2015/835985

**Published:** 2015-12-02

**Authors:** Christine J. Pol, Melissa Lieu, Konstantinos Drosatos

**Affiliations:** Temple University School of Medicine, Department of Pharmacology, Center for Translational Medicine, Philadelphia, PA 19140, USA

## Abstract

Over 5 million people in the United States suffer from the complications of heart failure (HF), which is a rapidly expanding health complication. Disorders that contribute to HF include ischemic cardiac disease, cardiomyopathies, and hypertension. Peroxisome proliferator-activated receptors (PPARs) are members of the nuclear receptor family. There are three PPAR isoforms: PPAR*α*, PPAR*γ*, and PPAR*δ*. They can be activated by endogenous ligands, such as fatty acids, as well as by pharmacologic agents. Activators of PPARs are used for treating several metabolic complications, such as diabetes and hyperlipidemia that are directly or indirectly associated with HF. However, some of these drugs have adverse effects that compromise cardiac function. This review article aims to summarize the current basic and clinical research findings of the beneficial or detrimental effects of PPAR biology on myocardial function.

## 1. Introduction

Heart failure (HF) is a major health issue that is anticipated to affect over 8 million people by 2030 [1]. Ischemic cardiac disease, cardiomyopathies, and hypertension are major risk factors that eventually lead to HF. Moreover, various drugs, which are used for treating metabolic disorders, have been associated with HF. Specifically, the drug class of peroxisome proliferator-activated receptor (PPAR) agonists have come under great controversy for adverse effects on cardiac function. PPAR agonists are indicated to treat a variety of metabolic disorders, like diabetes and hyperlipidemias, via individual or combined activation of PPAR isoforms.

PPARs are members of the class II nuclear hormone receptor superfamily. The three PPAR isoforms, PPAR*α*, PPAR*γ*, and PPAR*δ*, respond to a wide variety of endogenous ligands such as steroids, retinoids, and cholesterol metabolites [2, 3]. All PPARs can be activated by numerous endogenous ligands such as saturated and unsaturated fatty acids [4–6]. PPARs heterodimerize with retinoid X receptors (RXR) and bind to cis-acting DNA elements, known as PPAR response elements (PPREs), which increases gene transcription.

**Figure 1:**
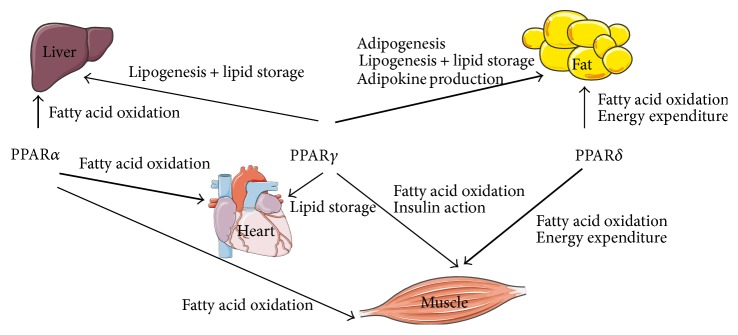
Metabolic regulation by PPARs. The different PPAR isoform regulates fatty acid and lipid metabolism in liver, heart, skeletal muscle, and adipose tissue. Figures were produced using Servier Medical Art (http://www.servier.com/).

PPAR*α*, PPAR*γ*, and PPAR*δ* regulate several aspects of lipid metabolism in the heart, skeletal muscle, liver, and adipose tissue (Figure 1). Tissue distribution of PPARs is broad [3]. PPAR*α* is primarily expressed in the liver but also present in the heart, intestine, adipose tissue, skeletal muscle, and kidney. PPAR*γ* is mainly expressed in adipose tissue and the large intestine and is a major regulator of adipocyte differentiation and storage. PPAR*δ* is expressed in all tissues.

This review aims to summarize basic and clinical research findings associating PPARs with beneficial or aggravating effects on myocardial function.

## 2. Transcriptional Regulation of PPARs

The transcription of PPARs can be regulated by multiple factors, such as pharmacological agents, hormone receptors, and fatty acids (Table 1). A marked reduction of cardiac PPAR*α* accompanies LPS administration [7, 8]. The mechanisms that lead to this reduction are not fully known. The JNK signaling pathway has been associated with reduced cardiac PPAR*α* gene expression [9]. Other factors such as HF [10], myocardial infarction (MI) [11], hypoxia [12, 13], IL-1*β* [14], IL-6 [14], PPAR*δ* [15, 16], NF-*κ*Β [17], glucose [18, 19], insulin [20], Akt [21], c-Myc [22], the Janus kinase/signal transducers and activators of transcription (JAK/STAT) pathway [23], reactive oxygen species [17], growth hormone [24], androgens [25], and angiotensin II [26] have also been reported to downregulate* Ppara* expression. There are several factors that are known to increase* Pparα* expression, such as glucocorticoids [27], farnesoid X receptor (FXR) [28], AMP-activated protein kinase (AMPK) [29–31], estrogen related receptor (ΕRR) *α* [32], retinoic acid [33], retinoid X receptor (RXR) [34], phorbol-12-myristate-13-acetate [35], exercise training [36], and heat shock factor-1 [37].* Ppara* gene expression levels and subsequent fatty acid oxidation (FAO) are upregulated by estrogen related receptor (ΕRR) *α*, which acts in conjunction with PPAR*γ* coactivator 1*α* (PGC1*α*) and binds directly to the PPAR*α* promoter [32].

PPAR*γ* is detected in several tissues and it is upregulated by various factors, such as C/EBPs [38, 39], estrogen [40], MEK/ERK signaling [41], c-Fos [42], TGF-*β* [43], Smad1 [44], p38 kinase, early growth-response factor-1 (Egr-1) [45], polyunsaturated fatty acids [19, 46, 47], the orphan nuclear receptor ROR*α* [48], the zinc-finger protein Zfp423 [49], and vitamin E [50]. Downregulation of PPAR*γ* is mediated by multiple factors including LPS [51, 52], JNK [53–55], TNF*α* [56–59], IL-11 [58], CCAAT/enhancer-binding protein homologous protein (CHOP) [60], retinoic acid [33, 61], estrogen receptor- (ER-) *α* [62], the JAK/STAT pathway [23, 38, 39], interferon-gamma [51, 63], leptin [64], angiotensin II [26], fasting [65], and androgens [66]. Krüppel-like factors (KLFs) have also been shown to affect PPAR*γ* and lipid metabolism in different ways. For instance, KLF5 [67] and KLF15 [68] induce PPAR*γ* expression and adipogenesis while KLF2 [53, 69] and KLF7 [70] have the opposite effect. KLF6 induces the transcription of PPAR*γ* and adipocyte differentiation [71], although it has been shown to cause the opposite effect as well [72].

PPAR*δ* plays a pivotal role in FAO, especially in adipose tissue and skeletal muscle. Similar to PPAR*α*, it is also induced by the AMPK-PGC1a axis and exercise training [73]. Other factors also increase* Ppard* expression such as promyelocyte leukemia (PML) tumor suppressor gene [74], extracellular-signal-regulated kinase 5 (ERK5) [75], hepatic lipase (HL) hydrolytic activity [76], LPS [77], and HIV-1 viral protein R (HIV-1 Vpr) [78]. PPAR*δ* mRNA levels increase after fasting and are returned to baseline with refeeding [79]. Other variables that downregulate PPAR*δ* expression are IL-6 [80], NF-*κ*B [81], and adipose triglyceride lipase (ATGL) deficiency [82]. In conclusion, PPARs are responsive to a wide variety of signals, which makes their biology complex.

## 3. Posttranslational Regulation of PPARs

PPARs undergo a number of posttranslational modifications that alter their activity. Regulation through phosphorylation, small ubiquitin-like modifier (SUMOylation), ubiquitination, O-GlcNAc modification, and acetylation have been documented.

### 3.1. Phosphorylation

PPAR*α* and PPAR*γ* activity can be modulated by phosphorylation. PPAR*α* and PPAR*γ* can be phosphorylated at serine residues by ERK/MAPK, protein kinase A (PKA), protein kinase C (PKC), AMPK, JNK, glycogen synthase kinase 3 (GSK3), and cyclin-dependent kinase 5 (Cdk5) [83, 84]. Phosphorylation by each of these kinases results in a differential modification of protein activity, which is dependent on the isoform, phosphorylation site, and cellular state [83]. PPAR*γ* phosphorylation at Ser273 by Cdk5 is blocked by PPAR*γ* agonists and decreased phosphorylation of PPAR*γ* at the Cdk5 site correlates with improved insulin sensitivity [84]. Contrary to what would be expected, adipose-specific Cdk5 knock-out mice (Cdk5-FKO) showed increased PPAR*γ* Ser273 phosphorylation and impaired glucose homeostasis despite unchanged food intake and body weight as wild type mice [85]. It was found that PPAR*γ* Ser273 is phosphorylated by both Cdk5 and ERK and Cdk5 inhibits the MEK/ERK pathway. Further inhibition of the ERK pathway improved glucose and insulin tolerance in the Cdk5-FKO mice [85]. PPAR*γ* transcriptional activation also decreases with phosphorylation. The S84A mutation increased PPAR*γ* activity as measured with a luciferase reporter system [86]. An example of PPAR phosphorylation leading to transcriptional activation is seen with insulin and fatty acid stimulation. A previous* in vitro* study showed that insulin increases PPAR*α* phosphorylation [87]. In addition to insulin, PPAR*α* phosphorylation could also be increased in rat adipocyte cultures treated with vanadate, an insulin mimetic, and okadaic acid. Increased PPAR*α* phosphorylation translated into an increase in PPAR*α* transcriptional activity. Although PPAR*δ* phosphorylation has not been studied to the same extent, this isoform contains consensus sites that have been predicted as potential targets of phosphorylation. Nevertheless, PPAR*δ* transcriptional activity is modulated by activation or inhibition of kinases, such as PKA [88] and p38 MAPK [89].

### 3.2. Ubiquitination and SUMOylation

Ubiquitin is a posttranslational modifier most known for its role in the nonlysosomal proteolytic pathway. A variety of proteins can be degraded through the ubiquitin system including PPARs [90]. Residues on PPAR*γ* that have been shown in literature to be targets for ubiquitination include K184 and K185 in adipocytes [90]. SUMO is a covalently bound posttranslational modification that is associated with a repression of PPAR activation [91–93]. SUMOylation occurs on lysine residues of all three PPAR isoforms [91, 94]. Reported SUMOylation sites include K185 for PPAR*α* in COS-7 and human hepatoma cells (HuH-7); K358 in NIH3T3 and HepG2 cells; K77, K107, K365, and K395 for PPAR*γ* in human embryonic kidney 293 (HEK293), HepG2, and NIH3T3 [92]; and K185 for PPAR*δ*. Although there is evidence that PPARs can be regulated by ubiquitin and SUMO in several cell types, there are limited studies in cardiomyocytes or cardiac tissue. Rodriguez et al. showed that increased activity of muscle ring finger-1 (MuRF1), a ubiquitin ligase, reduced PPAR*α* activity and FAO in neonatal rat cardiomyocytes (NRCMs) [95]. MuRF1 mediates monoubiquitination of PPAR*α* at residues K292, K310, and K358 which leads to nuclear export. MuRF1 did not target PPAR*δ* or PPAR*γ*, but other ubiquitin ligases may mediate ubiquitination of these isoforms.

### 3.3. O-GlcNAc Modification

O-GlcNAc transferase (OGT) catalyzes the addition of N-acetylglucosamine (O-GlcNAc) to serine or threonine residues of target proteins [96, 97]. O-GlcNAcase (OGA) catalyzes the removal of O-GlcNAc [97]. OGT modifies PPAR*γ* predominantly at Thr54 but not PPAR*α* or PPAR*δ* [97]. Inhibition of OGA blocked removal of O-GlcNAc, decreased PPAR*γ* transcriptional activity and adipogenesis, and inhibited insulin signaling [98]. As there are studies denoting O-GlcNAcylation by a cardiovascular stress signal, this type of modification of PPAR is emerging as a potential therapeutic target [96].

### 3.4. Acetylation

Acetylation refers to the addition of an acetyl group onto lysine residues of a substrate, which is catalyzed by histone acetyltransferases (HATs) and can be reversed by histone deacetylases (HDACs) [99].

Acetylation can occur on many proteins, including PPARs. It has been shown that HDAC3 interacts with PPAR*γ* and represses its activity [100]. Interaction between HDAC3 and PPAR*γ* is facilitated by retinoblastoma protein (RB), which binds both [101]. HDAC3 is present in the heart and is involved in cardiac energy metabolism. Mice with cardiomyocyte-restricted deletion of HDAC3 (*Hdac3cko*) showed modest upregulation of genes involved in FAO such as acyl-CoA oxidase 1 (AOX) and PDK4, which are PPAR responsive genes, without concomitant changes in PPAR gene expression levels [102]. However, the acetylation state of PPARs was not elucidated in this study. Determining how acetylation regulates PPARs in the heart would be advantageous for understanding how this posttranslational modification may modulate PPAR activity.

## 4. Gene Regulation by PPARs

PPARs bind to PPREs of genes that encode for fatty acid metabolism, inflammation, and adipocyte differentiation proteins. In the early 1990s, one of the first pieces of evidence that linked PPAR isoforms and FAO was found; it was shown that PPARs, particularly PPAR*α*, upregulate acyl-CoA oxidase, which catalyzes the first step in fatty acid *β*-oxidation [103]. Further studies have provided additional evidence that PPARs are master regulators of fatty acid metabolism.

Cardiomyocyte PPAR*α*, which is activated by intracellular TG-derived fatty acids [82, 104], regulates genes that encode for FAO-related enzymes like cluster of differentiation (*Cd*) 36, carnitine palmitoyl transferase I (*Cpt1*), diacylglycerol acyltransferase (*Dgat*), malonyl-CoA decarboxylase (*Mcd*), and fatty acid-binding protein (*Fabp*) [105]. Mice lacking PPAR*α* have reduced levels of FAO, increased glucose oxidation, and increased hepatic lipid content [106]. On the other hand, overexpression of PPAR*α* increases FAO and decreases glucose oxidation, while also surprisingly leading to cardiac lipid accumulation [107]. Cardiac-specific overexpression of PPAR*α* mice (*α*MHC-PPAR*α*) increases oxidation rate, measured through increased palmitate turnover from triacylglyceride (TAG) stores [108]. PPAR*α* activation can also increase cellular fatty acid uptake through CD36 and mitochondrial fatty acyl-CoA import via upregulation of* Cpt1* gene expression [109]. It was recently found that KLF15 and PPAR*α* cooperate synergistically to induce gene expression [110]. In conclusion, PPAR*α* plays a central role in controlling FAO and fatty acid uptake.

PPAR*γ* is vital for the regulation of adipogenesis and therefore is expressed in both white and brown adipose tissue, as well as in 3T3-L1 cells [111]. Target genes include adipocyte fatty acid-binding protein (aP2), CD36, lipoprotein lipase (LPL), phosphoenolpyruvate carboxykinase (PEPCK), and glucose transporter type 4 (GLUT4) [112]. Although PPAR*γ* is not as highly expressed in cardiac tissue as PPAR*α*, it is still critical for cardiac function. Four- and 8-month-old mice overexpressing PPAR*γ*_1_ (*α*MHC-PPAR*γ*1H) showed increased expression of downstream targets: CPT1, CD36, FA synthase (FAS), and adipose differentiation-related protein (ADRP) [113]. GLUT4 and GLUT1 were also upregulated in *α*MHC-PPAR*γ*1H. Hearts from *α*MHC-PPAR*γ*1H displayed an enlarged and dilated phenotype with decreased fractional shortening compared to controls, suggesting that PPAR*γ* influences cardiac remodeling.

Similar to PPAR*α*, PPAR*δ* is a regulator of FAO. PPAR*δ* is an important activator of genes involved in FAO in adipocytes and myocytes [79, 114]. Cardiomyocyte-specific knockout PPAR*δ* mice (CR-*Ppard* ^−/−^) displayed up to 50% decrease in FAO genes including* Cpt1*, long-chain acyl-CoA dehydrogenase (*Lcad*), 3-oxoacyl-CoA thiolase (thiolase), and pyruvate dehydrogenase kinase 4 (*Pdk4*) [115]. Reduced basal FAO in hearts from CR-*Ppard* ^−/−^ was associated with hypertrophy, dilation, and increased fibrosis [115]. Further, PPAR*δ* has a protective effect against high-fat-diet-induced obesity [114].

## 5. PPAR Animal Models

### 5.1. PPAR*α*

Genetic mouse models show the importance of PPAR*α* for the heart (Table 2). It has been well established that PPAR*α* ^−/−^ mice have decreased myocardial fatty acid metabolism [116–118]. Nevertheless, these mice have normal cardiac function at baseline according to several studies [118–120]. However, others have reported that PPAR*α* ^−/−^ mice have reduced cardiac function at baseline, which has been associated with fibrosis [117, 121], increased number of cristae in the mitochondria, increased number of caveolae in endothelial cells in the myocardium [117], and increased oxidative stress [122, 123]. Oxidative stress was caused by decreased MnSOD activity, and antioxidant therapy prevented left ventricular dysfunction, indicating that oxidative damage contributes to the cardiac dysfunction seen in mice that lack PPAR*α* [123]. These cardiac abnormalities progressed during aging [117]. PPAR*α* ^−/−^ mice also have an impaired response to metabolic stress. Following starvation, high temperature stress, and high workload, PPAR*α* ^−/−^ mice had lower levels of cardiac ATP [117, 120]. High workload challenge also decreased contractile performance [120]. Stimulation of *β*_1_-adrenergic receptors by isoproterenol resulted in reduced positive inotropic effect [121]. Short term starvation [106, 119] and CPT1 inhibition [116] caused hepatic and cardiac lipid accumulation and hypoglycemia. CPT1 inhibition also increased mortality.

Tg-PPAR*α* mice have mild cardiac hypertrophy, systolic dysfunction, and lipotoxicity, and over 50% die within 30 weeks [124, 125]. Cardiomyocyte-specific overexpression of PPAR*α* increases FAO and decreases glucose uptake and oxidation [107]. Together with ventricular hypertrophy and dysfunction, these mice have a phenotype similar to diabetic cardiomyopathy, since they have profound accumulation of intramyocardial triglycerides after short term fasting [107].

These studies implicate that PPAR*α* is important for activation of cardiac FAO and inhibition of glucose utilization. It is possible that PPAR*α* ^−/−^ mice do not always present with explicit cardiac dysfunction at baseline, because of an upregulation of glucose utilization [119]. However, this compensation is not sufficient during myocardial stress.

### 5.2. PPAR*γ*

Both transgenic and knockout PPAR*γ* mouse models have been generated (Table 2). Global PPAR*γ* ^−/−^ is lethal and the embryos have cardiac abnormalities caused by placental defects [126]. Cardiomyocyte-specific PPAR*γ* ^−/−^ mice develop cardiac hypertrophy with preserved systolic cardiac function and most likely have normal cardiac metabolism [127–129]. Increased NF*κ*B expression [127] or macrophage infiltration [128] might contribute to the development of hypertrophy. Isolated cardiomyocytes from PPAR*γ* ^−/−^ mice have increased length, which may also contribute to the observed hypertrophy [130]. A more severe phenotype was also found in cardiomyocyte-specific PPAR*γ* ^−/−^ mice [131]. These mice have increased oxidative damage. Beginning at 3-4 months of age, they develop progressive cardiac hypertrophy and mitochondrial abnormalities and eventually die from dilated cardiomyopathy [131]. Antioxidant treatment largely prevented pathological changes. PPAR*γ*-related gene expression profile was not changed in these models of PPAR*γ* ^−/−^, possibly due to compensatory mechanisms that may involve other PPAR isoforms. Inducible cardiomyocyte-specific PPAR*γ* ^−/−^ decreased expression of FAO-related genes and proteins and decreased FA utilization, whereas glucose utilization was not changed [132]. This led to only modest hypertrophy and reduced cardiac function. Mice with cardiomyocyte-specific PPAR*γ*1 overexpression have increased cardiac lipid accumulation, distortion of mitochondrial contours, disrupted cristae, and dilated cardiomyopathy. The timing and severity of the phenotype were dependent on the level of PPAR*γ* expression [113].

### 5.3. PPAR*δ*

PPAR*δ* in the cardiovascular system is of increasing interest and there are a number of mouse models that have been generated to study its role (Table 2). Total PPAR*δ* ^−/−^ results in embryonic lethality [133, 134]. Cardiomyocyte-specific PPAR*δ* ^−/−^ results in decreased FAO and increased glucose oxidation, cardiac lipid accumulation, hypertrophy, and fibrosis [115, 119]. Furthermore, these mice have mitochondrial abnormalities, develop dilated cardiomyopathy, and have reduced survival [115, 119]. Inducible cardiomyocyte PPAR*δ* ^−/−^ results in cardiac dysfunction associated with oxidative damage and mitochondrial abnormalities and cardiac hypertrophy [119, 135]. Interestingly, although cardiac dysfunction progressed over time, it did not decrease survival [135].

Meanwhile, cardiomyocyte-specific PPAR*δ* overexpression increased glucose utilization and glycogen content, while FA utilization remained normal. These mice do not develop cardiac lipid accumulation and have normal cardiac function [136]. Similarly, inducible cardiomyocyte-specific overexpression of constitutively active PPAR*δ* also increases glucose utilization [137]. However, these mice also have increased FAO and decreased glycogen content. Further, they have increased mitochondrial DNA content and increased mitochondrial biogenesis without oxidative stress and increased cardiac performance [137].

### 5.4. Animal Models with Combined Activation or Inhibition of PPAR Isoforms

The PPAR isoforms have overlapping functions and combined activation or inhibition of PPAR isoforms could aggravate or benefit the cardiac function. Cardiac dysfunction induced by cardiomyocyte-specific PPAR*γ* overexpression can be improved by PPAR*α* ^−/−^, although mice still have increased FAO and profound lipid accumulation [138]. Lipid redistribution and decreased mitochondrial and ER stress might contribute to the improved cardiac function and survival. In cardiomyocyte PPAR*δ* ^−/−^ mice, treatment with the PPAR*α* agonist fenofibrate increased* Cd36* and* Cpt1* gene expression but did not affect myocardial lipid content [129].

Cardiac dysfunction induced by cardiomyocyte-specific PPAR*δ* ^−/−^ could neither be rescued by PPAR*α* ^−/−^ nor worsen the phenotype compared to PPAR*δ* ^−/−^ [119]. The double PPAR*δ* ^−/−^; PPAR*α* ^−/−^ did not further decrease FAO; neither did it alleviate mitochondrial abnormalities, oxidative stress, hypertrophy, and cardiac dysfunction that was observed in the cardiomyocyte-specific PPAR*δ* ^−/−^.

Although the study of Bedu et al. mainly focuses on skeletal muscle, their study shows that double knockout of PPAR*α* and PPAR*δ* does not affect heart weight. Cardiac HAD activity, reflecting *β*-oxidation activity, is decreased only in the PPAR*α* ^−/−^ but is unchanged in the PPAR*δ* ^−/−^ or the double knockout [139]. This suggests that PPAR*δ* ^−/−^ can rescue decreased FAO in PPAR*α* ^−/−^. Further, cardiac citrate synthase (KREBS cycle activity) or LDH (glycolysis) activities are not changed in either the single or double knockout mice. Suggesting that PPAR*δ* ^−/−^ have unchanged cardiac metabolism and PPAR*α* ^−/−^ have decreased FAO that can be rescued by PPAR*δ* ^−/−^, in contradiction to other reports [115, 119].

Long-term treatment of rats with the pan-PPAR agonist tetradecylthioacetic acid (TTA) changes FA composition, including a decrease in saturated fat and arachidonic acid and an increase in n-3 PUFA [140]. Treatment of mice with TTA for 8 days increased FAO and decreased glucose oxidation, increased myocardial contractility, and reduced cardiac efficiency [141]. These effects appeared to be mediated via PPAR*α* since there was no effect of TTA treatment in PPAR*α*-null mice. Treatment of diabetic mice with the dual-PPAR*α*/*γ* agonist GCP-02 increased cardiac triglyceride content [142]. Treatment of* db/db* mice with the dual-PPAR*α*/*γ* agonist aleglitazar increased heart weight, whereas the PPAR*α*/*δ* agonist GFT 505 had no effect on heart weight [143]. Moreover, long-term treatment of cynomolgus monkeys had no adverse cardiac effects [143]. Treatment of rats with the dual-PPAR*α*/*γ* agonist LY510929 induced cardiac hypertrophy [144].

## 6. Cardiac Pathology: Involvement of PPAR Isoforms in Protection

**Figure 2:**
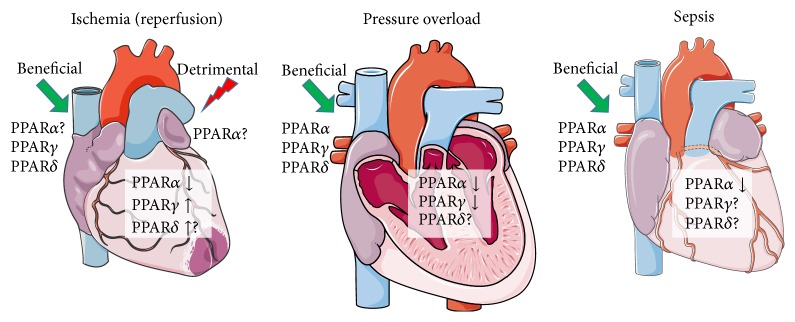
Effect of PPAR activation during cardiac dysfunction. Administration of PPAR agonists has generally been found to have beneficial effects on cardiac function during ischemia (with reperfusion), pressure overload induced hypertrophy, and sepsis-induced cardiac dysfunction. However, the role of PPAR*α* activation in ischemia reperfusion (I/R) injury is unclear as both beneficial and detrimental effects have been reported. Figures were produced using Servier Medical Art (http://www.servier.com).

**Figure 3:**
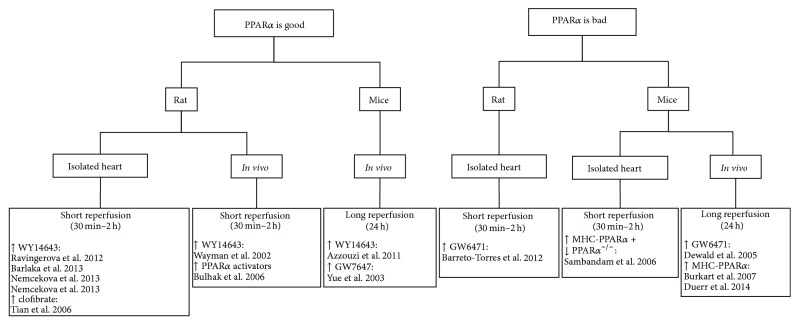
Effect of PPAR*α* activation on cardiac function after I/R. The role of peroxisome proliferator-activated receptor (PPAR) *α* activation in I/R injury is unclear as both beneficial and detrimental effects have been reported depending on the experimental model and timing of activation.

Several pharmacologic approaches aiming to either activate or inhibit PPARs have been used for treating various complications of cardiac function (Figure 2). PPAR agonist treatment is mostly beneficial in animal models of heart failure, but the beneficial or aggravating role of PPAR*α* activation in ischemia/reperfusion remains controversial (Figure 3).

### 6.1. PPAR*α*

#### 6.1.1. Aging-Related Cardiac Dysfunction

Cardiac PPAR*α* levels are decreased during aging [36, 145]. PPAR*α* ^−/−^ mice have decreased longevity [146]. Although this study did not find enhanced cardiomyopathy in the PPAR*α* ^−/−^ mice, minimal myocardial mineralization occurred more frequently in these mice. Metabolomic analysis showed an age-dependent decrease in cardiac glucose content and signs of decreased ketone supply and altered FA synthesis [147]. The cardiac abnormalities found in PPAR*α* ^−/−^ mice progressed as they aged [117].

Treatment of 20-month-old rats with the lipid-lowering drug atorvastatin increases PPAR*α*, PPAR*δ*, and PPAR*γ* expression [148]. Atorvastatin reduced cardiac hypertrophy, collagen deposition, oxidative stress, expression of inflammatory cytokines, and the aging marker *β*-galactosidase in aged rats. PPARs are known to have an anti-inflammatory effect [149, 150]. Pretreatment with PPAR inhibitors attenuated the inhibitory effect of atorvastatin on the expression of inflammatory cytokines, suggesting that part of the beneficial effects of atorvastatin on cardiac aging may be mediated by inhibition of inflammatory cytokines via PPAR signaling [148]. Another study also shows that activation of PPAR*α* in aged mice reduces inflammation [145].

#### 6.1.2. Pressure Overload Cardiac Hypertrophy

Most studies show decreased PPAR*α* after pressure overload induced cardiac hypertrophy. PPAR*α* levels are decreased at 1 week [151, 152], 9 days [153], and 4 weeks after aortic constriction [154, 155]. However, increased PPAR*α* levels at 4 weeks after aortic constriction have been reported as well [124].

Several studies show that treatment with the PPAR*α* agonist fenofibrate improves LV hypertrophy and remodeling after pressure overload in mice and rats. Treatment of mice with fenofibrate decreased hypertrophy, improved cardiac contractility, and decreased LV dilation at 4 weeks after transverse aortic constriction [154] and at 8 weeks after ascending aortic constriction [156]. Treatment of rats with fenofibrate for 4 weeks after abdominal aortic constriction decreased hypertrophy and fibrosis [155, 157]. Fenofibrate prevented the translocation of NFATc4 and p65 from cytoplasm to nucleus induced by pressure overload [155]. Fenofibrate treatment of spontaneously hypertensive rats (SHR) decreased hypertrophy, fibrosis, and oxidative stress in young SHR with cardiac hypertrophy. On the contrary, fenofibrate aggravated hypertrophy, fibrosis, and oxidative stress in old SHR with cardiac hypertrophy and decreased FAO [158]. PPAR*α* agonist WY14643 treatment of rats with cardiac hypertrophy and preserved cardiac power after ascending aortic constriction prevented energy substrate switching but decreased cardiac power [152].

Four weeks after TAC, mice displayed increased hypertrophy and decreased cardiac contractility in PPAR*α* ^−/−^ mice compared to wild type mice [159]. Additionally, hypertrophic, fibrotic, and inflammatory markers were higher in PPAR*α* ^−/−^ mice [159, 160]. Contrary, PPAR*α* ^+/−^ mice have less hypertrophy and less systolic dysfunction after TAC [124].

#### 6.1.3. Myocardial Ischemia

PPAR*α* expression is decreased at 4 weeks after MI in mice [161], increased at 6 weeks after MI in rats [162], and unchanged at 20 weeks after MI in rats [163]. Treatment of rats with a PPAR*α* agonist from 8 to 12 weeks after MI increased LV hypertrophy but did not worsen or improve cardiac function [163]. Treatment of rats that underwent MI with PPAR*α* agonist AVE8134 for 10 weeks after MI decreased fibrosis and improved cardiac function [164]. Thus, cardiac* Ppara* downregulation seems to constitute the initial response to MI, which reverses at a later stage. This may indicate an increased post-MI metabolic state in other cardiac cell types, such as fibroblasts.

#### 6.1.4. Ischemia/Reperfusion Injury

In isolated perfused rat hearts with 30 minutes of ischemia followed by 2 hours of reperfusion, the PPAR*α* agonist WY14643 or clofibrate improved cardiac contractile function and decreased infarct size [165–169]. In isolated perfused rat hearts with 30 minutes of ischemia followed by 30 minutes of reperfusion, the PPAR*α* inhibitor GW6471 blocked the beneficial effects of metformin in terms of cardiac contractility and mitochondrial function but had no detrimental effect by itself [170]. Beneficial effects on infarct size and cardiac performance were also found in rats and mice with* in vivo* ischemia reperfusion and PPAR*α* agonist treatment [171–175].

On the other hand, several studies reported detrimental effects of PPAR*α* after ischemia reperfusion. Isolated hearts from mice with cardiomyocyte-specific overexpression of PPAR*α* subjected to 18 minutes of ischemia followed by 40 minutes of reperfusion had decreased cardiac power associated with increased FAO and decreased glucose oxidation [176]. The opposite phenotype was found in hearts from PPAR*α* ^−/−^ mice. Also* in vivo* studies report increased infarct size and decreased cardiac function after ischemia followed by 24 hours of reperfusion with PPAR*α* agonist treatment [177] or in mice with cardiomyocyte-specific overexpression of PPAR*α* [136, 178]. Treatment of mice with the pan-PPAR agonist TTA for 8 days reduced recovery after I/R as indicated by a significant decrease in postischemic recovery of aortic flow, cardiac output, and rate-pressure product [141]. These effects are mediated by PPAR*α* since there was no effect of TTA treatment in PPAR*α*-null mice. Thus, activation of PPAR*α* during I/R may be either beneficial or detrimental, most likely determined by the timing of activation.

#### 6.1.5. Septic Cardiac Dysfunction

During sepsis, both inflammation and reduced FAO lead to cardiac dysfunction. A metabolomics study on sepsis patients showed an association between increased FAO markers and improved survival, suggesting that FAO is a potential therapeutic target [179]. Cardiac PPAR*α* expression is decreased within the first 24 hours after LPS-induced sepsis [7, 180]. Inducible cardiomyocyte-specific peroxisome proliferator-activated receptor *γ* coactivator 1-beta (PGC1*β*) overexpression largely reversed the LPS-mediated decrease of PPAR*α* expression and cardiac function [180]. Also, inhibition of JNK prevented the LPS-induced downregulation of PPAR*α*, FAO, and cardiac dysfunction [181]. However, treatment with PPAR*α* agonist could not prevent the LPS-induced cardiac dysfunction, likely due to profound inhibition of* Ppara* gene expression [182].

### 6.2. PPAR*γ*

#### 6.2.1. Pressure Overload Cardiac Hypertrophy

Treatment of mice with PPAR*γ* agonist pioglitazone from 1 week before until 3 weeks after abdominal aorta constriction decreased hypertrophy [183]. Pressure overload-mediated cardiac hypertrophy was more marked in PPAR*γ* ^−/+^ mice compared to wild type mice. Treatment with pioglitazone was less effective in these mice, implicating that the protective effect of pioglitazone is through PPAR*γ*. Pioglitazone treatment also decreased LV hypertrophy and fibrosis in Dahl salt-sensitive rats without lowering blood pressure [184]. The beneficial effects were associated with increased serum adiponectin and increased phosphorylation of AMPK in the heart, which indicate elevated cardiac FAO.

Mice have decreased PPAR*γ* expression after TAC, which is reversed in mice when TGF*β* signaling is blocked [185]. Treatment of mice with rosiglitazone from 3 days before till 3 weeks after TAC decreased fibrosis and hypertrophy, whereas treatment with PPAR*γ* antagonist had the opposite effect [185]. In rats with L-NAME induced hypertension, treatment with L-carnitine normalizes hypertension, hypertrophy, fibrosis, PPAR*γ* expression, and expression of fibrotic factors [186]. PPAR*γ* negatively correlates with fibrosis in these rats, suggesting that L-carnitine at least partly acts through PPAR*γ* activation. Thus, cardiac PPAR*γ* activation is protective against pressure overload hypertrophy.

#### 6.2.2. Myocardial Ischemia

Rats receiving PPAR*γ* agonist rosiglitazone from 6 hours to 8 weeks after MI had partially preserved LV function, but treatment did not prevent LV dilatation or hypertrophy. Moreover, it increased mortality [187]. However, treatment of mice with MI with PPAR*γ* agonist rosiglitazone from 3 days before till 1 or 2 weeks after MI resulted in decreased infarct size, apoptosis, and oxidative stress and improved cardiac function and survival [188]. Treatment increased adiponectin levels and the protective effects were absent in adiponectin knockout mice, suggesting PPAR*γ*'s protective effect is mediated by adiponectin.

Telmisartan, an AngII type I receptor blocker that also acts as partial PPAR*γ* agonist, was administered to rats with MI with improved LV remodeling and survival [189]. Although infarct size was not affected, treatment resulted in the alleviation of LV dilatation, hypertrophy, fibrosis, apoptosis, inflammatory cell infiltration, and ejection fraction. All of these beneficial effects were abolished by treatment with a PPAR*γ* antagonist, implying that telmisartan improves LV remodeling after MI via PPAR*γ* activation. Treatment of mice with PPAR*γ* agonist pioglitazone from 6 hours till 4 weeks after MI did not affect infarct size or survival but improved cardiac function and decreased LV dilatation, hypertrophy, fibrosis, and inflammatory cytokines [190].

#### 6.2.3. Ischemia/Reperfusion Injury

Several PPAR*γ* agonists reduce infarct size in rats with 25 minutes of ischemia followed by 2 hours of reperfusion [173]. Rosiglitazone treatment of rats with 30 minutes of ischemia followed by 4 hours of reperfusion reduced infarct size; involvement of the NF*κ*B pathway was indicated [191]. However, a high dose of rosiglitazone before ischemia is not protective.

Inducible cardiomyocyte-specific PPAR*γ* ^−/−^ increased infarct size after 30 minutes of ischemia followed by 4 hours of reperfusion [192]. Treatment with PPAR*γ* agonist pioglitazone reduced infarct size in both wild type and PPAR*γ* ^−/−^ mice, suggesting that the beneficial effect of pioglitazone is PPAR*γ* independent. However, pioglitazone treatment also reduced infarct size in rabbits with ischemia followed by 48 hours of reperfusion [193]. This effect was prevented by treatment with PPAR*γ* antagonist, (PI)3-kinase inhibitor, or nitric oxide synthase inhibitor, but not by a mitochondrial KATP channel blocker.

#### 6.2.4. Septic Cardiac Dysfunction

Mice with cardiomyocyte-specific PPAR*γ* overexpression are protected from LPS-induced decreased FAO and cardiac dysfunction [182]. Also, PPAR*γ* agonist protected LPS-treated mice from decreased FAO and cardiac dysfunction [182]. PPAR*γ* agonist treatment did not prevent elevated cardiac TG content as the cardiomyocyte-specific PPAR*γ* overexpression did, but it prevented a decrease in mitochondrial number and size. None of these treatments decreased the inflammatory response in the heart [181, 182]. Also treatment with PPAR*γ* agonist has been shown to be protective in LPS-treated rats, as it decreased mean arterial pressure, increased heart rate, increased inflammatory markers TNF*α* and IL-6, and increased markers of cardiac injury lactic dehydrogenase (LDH) and creatine phosphokinase (CPK) [194, 195].

### 6.3. PPAR*δ*

#### 6.3.1. Pressure Overload Cardiac Hypertrophy

Inducible cardiomyocyte-specific constitutively active PPAR*δ* overexpression does not affect TAC-mediated hypertrophy but improves LV dilatation, LV function, fibrosis, and mitochondrial abnormalities [137]. These findings indicate the importance of cardiac PPAR*δ* as a therapeutic target for alleviating certain aspects of cardiac pathology during hypertrophy.

#### 6.3.2. Myocardial Ischemia

Treatment of rats with MI with PPAR*δ* agonist immediately after MI had no beneficial effect on LV function. Nevertheless, it reversed the shift from FAO to glucose oxidation and normalized increased RV hypertrophy and lung congestion [196]. Also in mice, treatment with PPAR*δ* agonist from 8 to 12 weeks after MI did not change LV function [197]. Thus, PPAR*δ* activation seems not to be beneficial for post-MI LV function.

#### 6.3.3. Ischemia/Reperfusion Injury

Cardiomyocyte-specific overexpression of PPAR*δ* resulted in reduced infarcted area after 30 minutes of ischemia and 24 hours of reperfusion [136]. This is in contrast to cardiomyocyte-specific overexpression of PPAR*α* and might be due to the increased glucose oxidation seen in *α*MHC-PPAR*δ* mice, but not in *α*MHC-PPAR*α* mice [136, 178]. Also in rats, the activation of PPAR*δ* by treatment with agonist GW0742 resulted in decreased infarct size after 25 minutes of ischemia and 2 hours of reperfusion [198]. Whether treatment was applied before ischemia or at the start of reperfusion did not affect the improvement. It was proposed that the beneficial effect is caused by activation of the AKT pathway and subsequent inhibition of GSK3*β* and NF-*κ*B and inflammation [198].

#### 6.3.4. Septic Cardiac Dysfunction

Cardiac PPAR*δ* expression is decreased at 4 and 16 hours after LPS-induced sepsis [7]. Another study reported increased PPAR*δ* at 6 hours after LPS-induced sepsis and unchanged PPAR*δ* at 12 and 24 hours [180]. LPS-induced cardiac dysfunction is worsened in PPAR*δ* ^−/−^ mice [199]. Contrarily, treatment with PPAR*δ* agonist GW0742 attenuated LPS-induced cardiac dysfunction and improved survival after cecal ligation and puncture-induced sepsis [199]. The PPAR*δ* activation was associated with suppression of inflammatory pathways [199].

## 7. PPAR Agonists on Cardiac Function in the Clinical Setting

PPARs have been pharmacologically targeted through PPAR agonists, as described in numerous studies previously. In general, PPAR agonist binding enhances its activity and increases downstream target transcription. There are four main classes of PPAR agonists: PPAR*α*, PPAR*γ*, PPAR*δ*, and dual PPAR agonists.

### 7.1. PPAR*α* Agonists: Fibrates

Fibrates, such as fenofibrate, bezafibrate, ciprofibrate, and clofibrate, are PPAR*α* agonists used clinically for treating dyslipidemias such as primary hypertriglyceridemia, combined hyperlipidemia, and primary hypercholesterolemia [200]. Fibrates are generally well tolerated upon administration and theoretically beneficial as lowering LDL can reduce cardiovascular-related mortality [200–202]. Fibrates are reported to either have no effect on or decrease the risk of HF [202, 203]. The ACCORD Study showed that type II diabetic patients currently taking simvastatin and given fenofibrate had no significant difference in the number of HF events [203]. An older double-blind study in men with coronary heart disease receiving gemfibrozil instead of placebo had a 23% reduced risk of having a nonfatal MI [204]. Thus, fibrates seem to contribute to preserving cardiovascular health by decreasing coronary events [202, 204].

### 7.2. PPAR*γ* Agonists: Thiazolidinediones

TZDs are a major class of PPAR*γ* agonists that include rosiglitazone, pioglitazone, and troglitazone. TZD binding to the PPAR*γ*:RXR is thought to prevent corepressor interactions, thus enhancing transcriptional activity [205]. They are indicated for type II diabetes and help to improve insulin sensitivity in adipose tissue, skeletal muscle, and liver either via increased adiponectin levels [206, 207] or via increased glucose uptake [205]. Despite these benefits, rosiglitazone and pioglitazone have come under massive controversy for their cardiovascular-related effects. The use of pioglitazone may also be associated with an increased risk of bladder cancer [208]. Troglitazone has been removed from the market since 2000 due to its hepatotoxicity [209, 210]. In 2003, a retrospective study that included 17 million patients and their prescriptions, pharmacy, provider, and facility claims concluded that TZD was associated with a 60% increased risk for HF due to direct cardiovascular effects or other indirect effects [211].

Compared to pioglitazone, rosiglitazone appears to be associated with a higher risk of HF and other cardiovascular events, like stroke and MI [212]. Another study on the correlation and causation of TZDs and HF reported increased risk (43%) of MI in patients treated with rosiglitazone, compared to 82 deaths in the control groups treated with metformin, sulfonylurea, insulin, and placebo [209]. A TZD consensus statement acknowledged a small increase in HF incidents in patients on rosiglitazone but concluded that patients and health care providers should simply be aware of the risks [213]. A meta-analysis of randomized trials using rosiglitazone treatment found an association between rosiglitazone and increased risk for MI [209]. The PROactive study and a meta-analysis of randomized trials showed that although treatment of diabetes patients with pioglitazone increases heart failure incidence, subsequent all-cause mortality, MI, or stroke is decreased [214, 215]. Compared to pioglitazone, rosiglitazone appeared to be associated with a higher risk of HF and other cardiovascular events like stroke and MI [212]. However, the RECORD trial showed that rosiglitazone treatment is associated with an increased risk for heart failure, but not for MI, stroke, or cardiovascular mortality [216, 217]. A 2010 AHA/ACCF Science Advisory reevaluated TZDs and their cardiovascular risks based on more recent clinical trials and meta-analyses and concluded that a link between rosiglitazone and HF could not be established [210]. In 2013 the FDA removed restrictions on rosiglitazone.

### 7.3. PPAR*δ* Agonists

PPAR*δ* agonists are neither as widespread nor as developed as PPAR*α* or PPAR*γ* agonists. Currently, telmisartan is one drug on the market that targets PPAR*δ*, as well as PPAR*γ* [218]. Telmisartan is indicated for hypertension, as it is an angiotensin II receptor blocker (ARB), but it can also partially target PPAR*δ* [218, 219]. HF is included in the list of spontaneous events most frequently reported during postmarketing surveillance, but it remains unknown how concrete the link between PPAR*δ* agonists and cardiac function is. A study that assessed the risk of cardiovascular events in patients, who recently suffered from an ischemic stroke, using telmisartan, showed a slightly less rate of developing MI and HF for the telmisartan group [220]. There have been two trials on the effects of telmisartan: ONTARGET and TRANSCEND [221]. The ONTARGET trial randomly divided 25,620 patients into three groups to receive telmisartan, ramipril, or a combination of both [222]. No significant differences were observed between the groups in terms of primary outcomes (fatal cardiovascular complications, MI, HF, or stroke) and secondary outcomes (revascularization, nonfatal HF, diabetes, angina, or renal impairment) [222]. The TRANSCEND trial, which utilized 6,000 patients receiving telmisartan or placebo, came to a similar conclusion [223]. However, the females that used telmisartan showed a 20% overall risk reduction of MI [221]. It is difficult to determine whether telmisartan's beneficial effect on cardiac function is accounted for by direct action of the drug on cardiac PPAR*δ* or solely because of ARB targeting.

### 7.4. Dual- and Pan-PPAR Agonists: Glitazars

The fourth class of PPAR agonists includes the dual-PPAR agonists and the pan-PPAR agonists, also known as glitazars. The insulin sensitizing effects of the PPAR*γ* agonists combined with the lipid-lowering effects of the PPAR*α* agonists would theoretically be efficacious in treating patients with metabolic syndrome or type II diabetes. Indeed, dual-PPAR*α*/*γ* agonists have been in development under great interest. Although there are none approved in the US, saroglitazar was approved in June 2013 for clinical use in India [224]. Saroglitazar has a higher affinity for PPAR*α* than PPAR*γ*. Saroglitazar, like the PPAR*α* agonists, is generally well tolerated and significantly effective (*P* < 0.001) in lowering plasma triglyceride levels, 45% reduction compared to control [225]. It is too early to tell whether saroglitazar has any cardiovascular impact, although its product information contains a warning and precautionary statement with its use in type II diabetics with congestive HF [226]. Saroglitazar is still in its Phase IV postmarketing surveillance study. Other glitazars that were in development include aleglitazar, muraglitazar, tesaglitazar, and cevoglitazar. As of present, all have been abandoned due to adverse side effects, including cardiovascular adverse effects. The trials evaluating aleglitazar, called AleCardio, were halted during Phase III trials in July 2013 due to increased incidents of gastrointestinal hemorrhage, bone fractures, and HF in patients receiving aleglitazar compared to placebo [227]. Similarly, muraglitazar, another dual-PPAR*α*/*γ* agonist, had a negative cardiovascular impact on its patients. In an analysis of multiple clinical trials, muraglitazar was compared to pioglitazone and placebo in order to assess the cardiovascular risks [228]. Muraglitazar, as monotherapy or as combination therapy, had higher incidents of HF, MI, and transient ischemic attacks (TIAs) compared to control. The mechanism of cardiovascular toxicity of these dual-PPAR*α*/*γ* agonists is still unknown and needs to be elucidated [227, 228].

## 8. Epilogue

PPARs have major roles in regulating cardiac metabolism and function in health and disease. Administration of PPAR agonists or antagonists can be either beneficial or detrimental for cardiac function depending on the type of stress that the heart undergoes and the timing of administration. Thus, alteration of PPAR activation may be used in therapeutic approaches that aim to improve cardiac function.

## Figures and Tables

**Table 1:** Transcriptional regulators of PPARs (see text for acronyms).

Target	Effect	Stimulus
PPAR*α*	↓	LPS [7, 8], JNK signaling [9], HF [10], MI [11], hypoxia [12, 13], IL-1*β* [14], IL-6 [14], PPAR*δ* [15, 16], NF-*κ*Β [17], glucose [18, 19], insulin [20], Akt [21], c-Myc [22], JAK/STAT pathway [23], ROS [17], growth hormone [24], androgens [25], and angiotensin II [26]
	↑	Glucocorticoids [27], FXR [28], AMPK [29–31], ERR*α* [32], retinoic acid [33], RxR [34], phorbol-12-myristate-13-acetate [35], exercise training [36], and heat shock factor-1 [37]

PPAR*δ*	↓	IL-6 [80], NF-*κ*B [81], and ATGL deficiency [82]
	↑	AMPK-PGC1a axis, exercise training [73], PML tumor suppressor gene [74], ERK5 [75], HL hydrolytic activity [76], LPS [77], HIV-1 Vpr [78], and fasting [79]

PPAR*γ*	↓	LPS [51, 52], JNK [53–55], TNF*α* [56–59], IL-11 [58], CHOP [60], retinoic acid [33, 61], ER-*α* [62], JAK/STAT pathway [23, 38, 39], interferon-gamma [51, 63], leptin [64] angiotensin II [26], fasting [65], androgens [66], KLF2 [53, 69], KLF7 [70], and KLF6 [72]
	↑	C/EBPs [38, 39], estrogen [40], MEK/ERK signaling [41], c-Fos [42] TGF-*β* [43], Smad1 [44], p38 kinase, Egr-1 [45], polyunsaturated fatty acids [19, 46, 47], the orphan nuclear receptor ROR*α* [48], Zfp423 [49], vitamin E [50], KLF5 [67], KLF15 [68], and KLF6 [71]

**Table 2:** PPAR mouse animal models.

Target	Model	Cardiac metabolism	Cardiac function	Reference
PPAR*α*	PPAR*α* ^−/−^	Defective lipid and glucose homeostasis		[116]
		Defective lipid homeostatic response to fasting		[106]
		Decreased FAO, abnormal mitochondria	Fibrosis, progressed during aging	[117]
		Decreased FAO, increased glucose oxidation and glycolysis	Normal cardiac function	[118]
		Substrate switch from fatty acid to glucose, inefficient ATP generation	Normal cardiac function	[120]
			Systolic ventricular dysfunction, fibrosis	[121]
			Increased oxidative stress, LV dysfunction	[122, 123]
		Decreased FAO, increased glucose oxidation	Normal cardiac function	[119]
	*α*MHC-PPAR*α*	Increased FAO, decreased glucose oxidation and uptake	Ventricular hypertrophy, systolic ventricular dysfunction	[107]

PPAR*δ*	PPAR*δ* ^−/−^		Impaired development	[134]
			Embryonic lethality	[133]
	*α*MHC-PPAR*δ* ^−/−^	Decreased FAO and increased glucose oxidation, lipid accumulation	Cardiac dysfunction, hypertrophy, and reduced survival	[115]
		Decreased FAO and normal glucose oxidation	Hypertrophy, mitochondrial abnormalities, and cardiac dysfunction	[137]
	Inducible *α*MHC-PPAR*δ* ^−/−^	Decreased FAO and glucose oxidation, mitochondrial abnormalities	Cardiac dysfunction, oxidative damage, and hypertrophy	[135]
	*α*MHC-PPAR*δ*	Normal FAO, increased glucose oxidation	Normal cardiac function	[136]
	Inducible *α*MHC-PPAR*δ*	Increased FAO and glucose oxidation, increased mtDNA	Enhanced cardiac contractility	[137]

PPAR*γ*	PPAR*γ* ^−/−^		Embryonic lethality	[126]
	*α*MHC-PPAR*γ* ^−/−^		Hypertrophy, preserved systolic function	[127]
			Hypertrophy, mitochondrial oxidative damage, and dilated cardiomyopathy	[131]
		No changes in cardiac metabolism at baseline		[129]
	Inducible *α*MHC-PPAR*γ* ^−/−^	Decreased FAO, normal glucose oxidation	Decreased cardiac contractility, modest hypertrophy	[132]
	MLC2v-PPAR*γ* ^−/−^		Hypertrophy, macrophage infiltration	[128]
	*α*MHC-PPAR*γ*1	Increased TG uptake, increased lipid and glycogen stores, and abnormal mitochondria	Dilated cardiomyopathy	[113]
	MLC2v-PPAR*γ*		Increased cardiomyocyte length	[130]
